# Study on the Effect of CTBN and h-BN Synergistic Toughening on the Damping Properties of Carbon-Fiber-Reinforced Epoxy Composites

**DOI:** 10.3390/polym18050578

**Published:** 2026-02-27

**Authors:** Wei Wang, Xueping Gao, Zhimin Li, Yishi Wang, Bo Zhu

**Affiliations:** 1 Carbon Fiber Research Engineering Center, School of Materials Science and Engineering, Shandong University, Jinan 250061, China; wangwei2023@mail.sdu.edu.cn (W.W.);; 2 Key Laboratory of Liquid-Solid Structural Evolution and Processing Materials of Ministry of Education, Shandong University, Jinan 250061, China

**Keywords:** CTBN, hexagonal boron nitride, epoxy, CFRP, damping, dynamic mechanical analysis, free-vibration, UD prepreg

## Abstract

Carbon-fiber-reinforced polymer (CFRP) composites possess outstanding specific stiffness and strength but typically exhibit low intrinsic damping, which limits vibration attenuation in lightweight dynamic structures. Herein, a hybrid toughening strategy combining carboxyl-terminated butadiene nitrile rubber (CTBN) and hexagonal boron nitride (h-BN) is developed to enhance the damping of CFRP laminates while preserving cure feasibility and thermomechanical stability. An E51/DICY/accelerator epoxy system (100:6.5:1.2, mass ratio) is used as the baseline matrix. Differential scanning calorimetry shows that both CTBN and h-BN shift the cure peak temperature upward (*Tp*: 160.6 → 170.3 °C) and reduce the reaction enthalpy (*ΔH:* 386.5 → 255.1 J/g), indicating dilution/transport effects and altered cure kinetics. Dynamic mechanical analysis (DMA) reveals that CTBN exhibits an optimum damping enhancement at 25 phr (*tan δ_max* = 0.300), whereas h-BN provides a stronger monotonic increase up to 25 phr (*tan δ_max* = 0.437). Notably, the CTBN/h-BN hybrid (25/25 phr) delivers a high *tan δ_max* of 0.468 together with the broadest effective damping window (*ΔT_half* = 28.6 °C), exceeding 85% of the linear additivity criterion proposed herein. When the materials are transferred into CFRP laminates, free-vibration tests (using the logarithmic decrement method) demonstrate a clear structural damping improvement (*ζ:* 0.021 → 0.035; *δ:* 0.132 → 0.221; t_1_/_2_: 0.48 → 0.27 s). Overall, the results suggest that the damping enhancement arises from a combination of EPBN-mediated ductile energy dissipation and h-BN-related interfacial/interlayer frictional losses, which can be jointly tuned to balance processability, thermal response, and damping performance in CFRPs.

## 1. Introduction

CFRP composites are widely used in aerospace, transportation, wind energy, and high-end sports equipment due to their light weight and high mechanical efficiency, but their low damping leads to slow vibration decay and potential comfort/safety issues in dynamic services [1,2,3]. In polymer–matrix composites, damping originates mainly from (i) intrinsic viscoelasticity of the matrix/reinforcement and (ii) interface-related dissipation near fiber–matrix or filler–matrix interfaces [2,3,4,5,6]. Rubber toughening (e.g., CTBN) can increase damping by introducing an elastomeric phase that activates additional relaxation and frictional losses, but it often reduces stiffness and the glass transition temperature (Tg), creating a damping–stiffness/thermal trade-off [7,8,9,10,11,12,13,14]. Layered fillers such as hexagonal boron nitride (h-BN) can enhance damping via interface-dominated mechanisms (e.g., stick–slip friction and potential interlayer sliding), often with a smaller stiffness penalty than rubber alone [5,14,15,16,17,18,19,20,21,22,23,24]. Although CTBN/BN hybrid systems have been explored in cast epoxy, it remains unclear how a processing-compatible CTBN/h-BN formulation (latent-cured E51/DICY) transfers its resin-stage damping gains into industrially relevant UD prepreg CFRP laminates, where fiber-dominated stiffness can dilute matrix viscoelastic losses.

From a vibration-control standpoint, the modal loss factor (or damping ratio) of a CFRP is often substantially lower than that of metals or high-loss polymers, which may lead to slow vibration decay, acoustic radiation, and comfort/safety issues in service. A broad range of damping-enhancement strategies have been reported, including viscoelastic interleaves/coatings, hybrid fiber architectures, and nano-/micro-scale fillers that activate interfacial frictional dissipation. Recent reviews emphasize that effective damping design in fiber composites is essentially a multi-objective optimization: increasing internal friction (loss factor) without disproportionally degrading stiffness/thermal stability or processability. Rubber toughening of epoxy (e.g., CTBN) is a classic way to increase fracture toughness via reaction-induced phase separation, where a dispersed elastomeric phase promotes cavitation and matrix shear yielding. Beyond toughness, CTBN can also increase damping by activating additional relaxation/friction processes; however, it commonly induces trade-offs such as reduced moduli and glass transition temperatures. For example, Mansour et al. reported that CTBN-modified epoxy exhibits enhanced damping capacity as measured via vibration/DMA measurements, accompanied by a notable stiffness penalty.

In contrast, two-dimensional layered fillers provide an interface-dominated dissipation pathway. In CFRP and related polymer composites, oriented multilayer sheets can trigger energy dissipation through interfacial stick–slip and internal interlayer sliding, leading to improved damping over a wide temperature/frequency range. These considerations motivate a hybrid “rubber + layered filler” design: CTBN contributes ductile dissipation and interface toughening, while h-BN contributes frictional/interlayer losses and can partially compensate for the thermal response. Hybrid BN/epoxy systems toughened by CTBN have also been discussed as a viable approach to balancing multifunctional properties.

In this study, we address the following research questions: (Q1) How do CTBN and h-BN individually influence the cure behavior, processability (viscosity/gel time), and DMA-based damping descriptors (*tan δ_max*, *T_tan δ*, and *ΔT_half*) of a latent-cured E51/DICY epoxy system? (Q2) Can a CTBN/h-BN hybrid matrix yield superior laminate-level damping (assessed via DMA and the free-vibration damping ratio ζ) realtiveto matrices modified with a single component while maintaining a realistic storage modulus benchmark for 0° unidirectional (UD) CFRP laminates? (Q3) Relative to a linear-additive reference model, what is the quantitative ‘synergy efficiency’ of the hybrid response when experimental uncertainty is accounted for? To answer these questions, the present work delivers (i) a processing-to-performance map linking DSC/rheology with DMA damping metrics for CTBN, h-BN, and their hybrids; (ii) a laminate-level validation using 0° UD prepreg CFRP (DMA and free-vibration); and (iii) a conservative, uncertainty-aware additivity analysis reported as a synergy-efficiency index rather than an arbitrary pass/fail rule.

## 2. Materials and Methods

### 2.1. Materials

This study employed an epoxy resin, E51 (bisphenol-A type epoxy, EEW 184–200 g /eq; Nantong Xingchen Synthetic Material Co., Nantong, China); a curing agent, dicyandiamide (DICY, H106; Shandong Usolf Chemical, Linyi, China); and a urea-based accelerator (F401; Changzhou Calla Resin Co., Ltd., Changzhou, China). The baseline formulation E51:H106: F401 = 100:6.5:1.2 (mass ratio) follows the supplier-recommended latent-curing window for E51/DICY systems and was further verified via preliminary DSC screening to ensure complete cure and a stable Tg while maintaining a practical prepreg-processing viscosity. The following materials were used: toughener—epoxy-terminated butadiene nitrile rubber (EPBN; Aladdin Reagent Co., Ltd., Shanghai, China); filler—hexagonal boron nitride (h-BN, platelet morphology, D50 ≈ 1–2 μm; Denka Chemicals Co., Ltd., Tokyo, Japan); and carbon fiber reinforcement—T700 12K unidirectional (UD) carbon-fiber/epoxy prepreg (FAW 100 g/m^2^; resin content, 37 wt%; 20 plies consolidated to ~2.0 mm thickness, Weihai Guangwei Group Co., Ltd., Weihai, China). All laminate specimens were cut and tested in the 0° (fiber) direction.

Rheology was measured using a cone–plate viscometer (Brookfield CAP2000+, Brookfield Engineering Laboratories, Inc., Middleboro, MA, USA) at 70 °C. Gel time was determined isothermally at 120 °C using a standard time-to-gel criterion (flow cessation) on the same viscometer. DSC was performed on a Mettler-Toledo DSC 3+ (METTLER TOLEDO Technology (China) Co., Ltd., Shanghai, China) under N2 (50 mL min^−1^) with 8–10 mg samples in sealed Al pans; non-isothermal scans were conducted from 30 to 250 °C at 10 °C min^−1^. DMA was conducted using a Mettler-Toledo DMA/SDTA 861e (METTLER TOLEDO Technology (China) Co., Ltd.) in single-cantilever mode for resin castings and dual-cantilever mode for laminates; temperature sweeps were performed from 30 to 200 °C at 3 °C min^−1^ and 1 Hz with a strain amplitude of 0.05%.

### 2.2. Synergy Criterion

Four matrix series are defined as shown in Table 1 (phr refers to parts per hundred resin, by mass):

Synergistic/Additivity Analysis. We performed a synergistic/additivity analysis by comparing the mixed response to a linear-additive reference model: *P_add_ = P_C_ + P_B_ − P_EP_*, where *P* represents the damping descriptor (e.g., *tan δ_max*). We report the synergistic efficiency exponent *η = P_hybrid_/P_add_* (*η* > 1 indicates super-additive behavior, *η* ≈ 1 indicates near-additive behavior, and *η* < 1 indicates sub-additive behavior). All DMA-derived descriptors are reported as means ± standard deviations (*n* = 3). When discussing superadditivity, we compare *P_hybrid_* and *P_add_* with their propagation uncertainties to ensure that any difference is greater than the experimental error.

### 2.3. Resin Preparation

E51 epoxy was preheated to 60–70 °C to reduce viscosity. CTBN (EPBN) was added under mechanical stirring until a visually homogeneous mixture was obtained. For h-BN-containing formulations, h-BN platelets were incorporated via high-shear mixing to minimize agglomeration. After cooling to <60 °C, DICY (H106) and the accelerator (F401) were added in the specified mass ratio (E51:H106:F401 = 100:6.5:1.2). The mixture was degassed under vacuum and cast into molds for DSC/DMA and adhesion testing. All samples were prepared at least in triplicate to evaluate repeatability.

### 2.4. CFRP Laminate Fabrication

UD prepreg laminates were fabricated by stacking 20 plies in a unidirectional lay-up all oriented at 0°and consolidating them in a hot press under vacuum bagging. The laminate thickness after consolidation was ~2.0 mm. A representative cure cycle for the E51/DICY system is as follows: 120 °C for 1 h (ramp 2 °C/min), followed by 160 °C for 2 h under 0.5–1.0 MPa pressure and then cooling to room temperature under pressure. Rectangular specimens were cut along 0° (fiber direction) for DMA and free-vibration tests.

### 2.5. Characterization

#### Characterization and Testing

DSC. Non-isothermal DSC was used to record cure exotherms and extract the peak temperature (*Tp*) and reaction enthalpy (*ΔH*). The glass transition temperature (*Tg*, DSC) of cured samples was obtained from the heat-capacity step [7,8,9]. Samples (8–10 mg) were heated from 30 to 250 °C at 10 °C min^−1^ under N_2_ (50 mL/min), with at least three repeats.

Rheology/processability: Viscosity at 70 °C was taken to be an “initial viscosity” indicator relevant to infusion/prepreg processing. Gel time was determined at 120 °C using the conventional time-to-gel criterion (rapid viscosity rise/flow cessation) [18]. Viscosity was measured at 70 °C with a cone–plate geometry (CAP2000+; spindle No., 6; shear rate, 100 /s) after 5 min of thermal equilibration. Gel time at 120 °C was taken to be the time when the apparent viscosity exceeded 10^5^ mPa·s.

DMA of neat resin castings: Temperature-sweep DMA was conducted to obtain storage modulus (E′), loss modulus (E″), and loss factor *tan δ*, defined as *tan δ* = E″/E′. The maximum loss factor (*tan δ_max*), the corresponding peak temperature (*T_tan δ*), and the half-peak breadth (*ΔT_half*, as an effective damping temperature window) were extracted for comparison [25]. Rectangular specimens (35 × 10 × 2 mm^3^) were tested in single-cantilever mode (1 Hz, 0.05% strain, 3 °C min^−1^, and 30–200 °C).

DMA of CFRP laminates: Laminates with different matrices were tested via temperature-sweep DMA to evaluate the matrix-to-laminate transfer of damping enhancement [5,6,24]. Laminate strips (50 × 10 × 2 mm^3^) were tested in dual-cantilever mode.

Free-vibration test and damping extraction. Cantilever beams were excited by an initial displacement/impact, and the decaying tip response was recorded. The logarithmic decrement *δ* was calculated from successive peak amplitudes: $\delta =\frac{1}{n}ln\left(\frac{x_{0}}{x_{n}}\right)$, *x*_0_ = amplitude of the first oscillation peak; *x_n_* = amplitude of the peak after *n* complete cycles. The damping ratio *ζ* was obtained from δ using the standard underdamped relationship $\zeta =\frac{\delta }{\sqrt{4\pi^{2}+\delta^{2}}})$ [9,10,12,17]. Cantilever beams (150 × 15 × 2 mm^3^; free length, 120 mm) were recorded with a laser displacement sensor (Keyence LK-G5000, KEYENCE Corporation, Osaka, Japan).

For viscoelastic damping benchmarking, the above approach is consistent with widely used vibration-based damping identification methods; in parallel, beam-based system methods (e.g., Oberst beam) are also commonly adopted for damping materials characterization [5,24,25,26]. Single-lap shear strength was measured according to ASTM D1002 [27]—overlap length, 12.5 mm; adhesive thickness, 0.2 ± 0.02 mm; and crosshead speed, 1.3 mm min^−1^ (T-peel: ASTM D1876 [28] crosshead speed, 254 mm min^−1^)—with at least five replicates.

## 3. Results and Discussion

### 3.1. Baseline Epoxy Formulation and Damping Metrics

The baseline matrix (E51/DICY/accelerator = 100:6.5:1.2) was selected to provide a controlled cure window and a moderate glass transition temperature suitable for further toughening/filler addition. In polymer composites, damping originates from (i) the intrinsic viscoelasticity of the matrix and (ii) interface-related dissipation (localized viscoelastic losses and friction near fiber/matrix or filler/matrix interfaces). Therefore, the DMA loss factor (tan *δ*) is widely used as a practical descriptor of thermomechanical damping, while free-vibration parameters (*δ*, ζ) quantify the structural vibration decay of laminate-level specimens.

In this work, three DMA-derived indicators are emphasized: *tan δ_max* (peak damping intensity), *T_tan δ* (service temperature location), and *ΔT_half* (effective damping temperature breadth). A broad *ΔT_half* is practically important because CFRP components are often subjected to varying thermal environments; thus, damping enhancement should be effective over a temperature interval rather than at a single point.

### 3.2. Effect of CTBN on Epoxy Curing Behavior, Processability, and Damping

**Figure 1:**
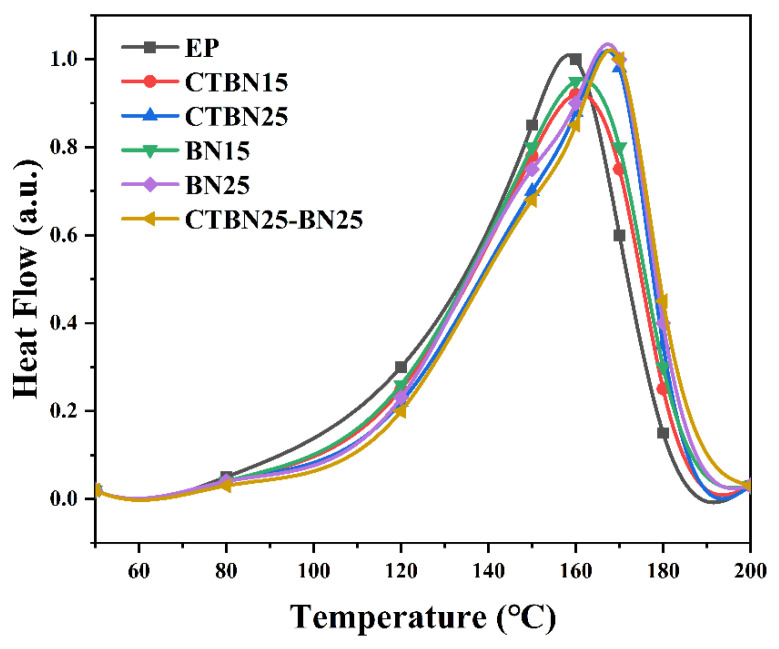
DSC curing curves of epoxy systems modified with CTBN and h-BN at different loadings.

Cure behavior (DSC). Figure 1 and Table 2 show that CTBN systematically increases the cure peak temperature (*Tp*) while reducing the overall reaction enthalpy (ΔH). For example, compared with neat epoxy (*Tp* = 160.6 °C; ΔH = 386.5 J g^−1^), CTBN-modified systems exhibit higher *Tp* and markedly lower ΔH (e.g., C25: *Tp* = 167.4 °C; ΔH = 264.6 J g^−1^). This behavior is consistent with a combined dilution/transport effect and reaction-induced microphase development, which can delay the attainment of the maximum reaction rate and broaden diffusion-controlled curing in later stages [11,12,29,30,31,32].

**Figure 2:**
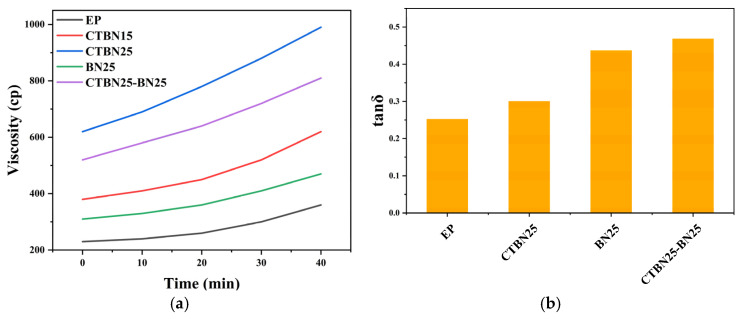
Processability evaluation of modified epoxy resins: (**a**) viscosity evolution at 70 °C and (**b**) gel time measured at 120 °C for different formulations.

From a manufacturing perspective, CTBN imposes a pronounced viscosity penalty, and Figure 2a shows a steep, non-linear viscosity increase as the CTBN loading increases, which can be associated with the development and coarsening of rubber-rich domains that impede flow. Gel time at 120 °C (Figure 2b) provides a practical processing-window indicator: the neat epoxy (EP) exhibits a gel time of ~18 min, whereas CTBN addition markedly prolongs gelation, which is primarily attributable to dilution of the reactive group-concentration and hindered molecular diffusion caused by the increased viscosity and emerging two-phase morphology. In contrast, the influence of h-BN on gel time is not pronounced, likely because its high thermal conductivity facilitates internal heat transfer and partially compensates for the mild diffusion limitation induced by particle-filled rheology. Therefore, combining CTBN with h-BN is practically meaningful: CTBN provides strong viscoelastic dissipation but extends gelation, while h-BN improves damping with a comparatively limited gel-time penalty, resulting in a more balanced processability–performance window.

**Figure 3:**
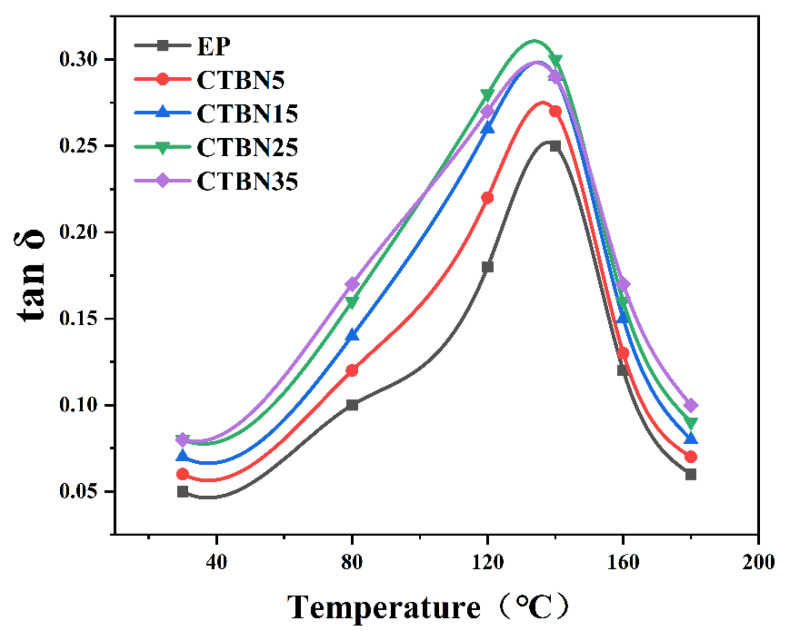
Temperature dependence of loss factor (*tan δ*) for CTBN-toughened epoxy resins at different CTBN contents.

Figure 3 and Table 3 demonstrate a non-monotonic CTBN effect: *tan δ_max* increases from 0.273 (C5) to 0.300 (C25) and then slightly decreases (0.291 at C35), while T_tanδ shifts downward (142.1 → 136.7 °C) and *ΔT_half* broadens (18.5 → 24.8 °C). The peak broadening indicates that CTBN activates additional relaxation/frictional dissipation pathways over a wider temperature range, whereas the mild decline at high CTBN loadings can be explained as phase coarsening and defect-like heterogeneity that reduce effective energy dissipation per unit strain. A similar CTBN-driven damping enhancement with stiffness trade-offs has been reported in epoxy/CTBN systems through DMA and vibration analysis.

### 3.3. Effect of h-BN on Damping and Thermomechanical Behavior (DMA)

**Figure 4:**
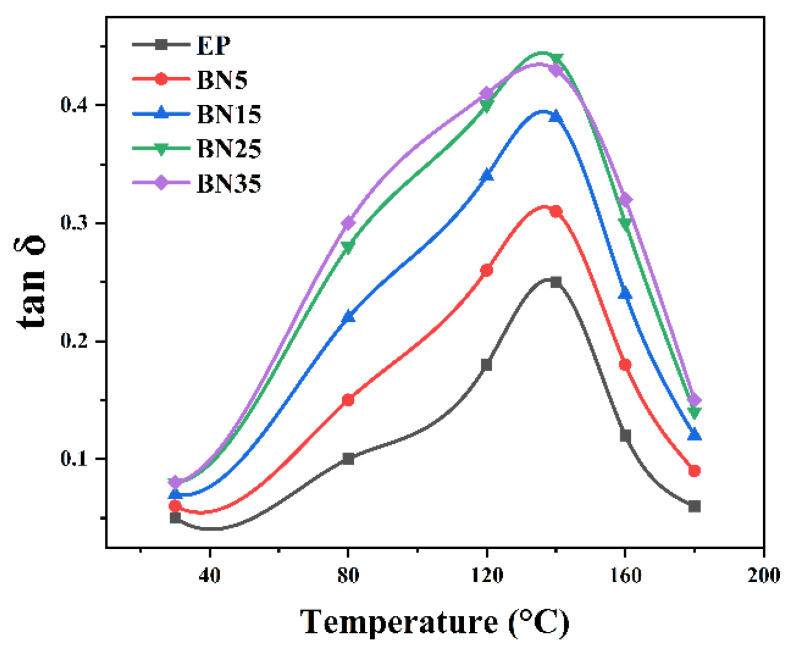
Temperature dependence of loss factor (*tan δ*) for h-BN-modified epoxy resins at different h-BN loadings.

Within the studied range, h-BN increases the loss factor in a largely monotonic manner: *tan δ_max* rises from 0.252 (EP) to 0.389 (B15) and 0.437 (B25). Figure 4 shows that the *tan δ* peak temperature changes only modestly relative to EP, indicating that h-BN mainly increases damping intensity rather than strongly shifting the relaxation temperature. The likely origin is interface-dominated dissipation associated with platelet-filled epoxies (e.g., interfacial friction/stick–slip and constrained-chain interphase), as widely discussed in the literature; however, in this study, these mechanisms should be regarded as plausible interpretations rather than direct experimental proof because no evidence of microstructural sliding was obtained (via, e.g., in situ tribology or interphase spectroscopy) [23,24].

### 3.4. Adhesion-Related Mechanical Indicators and Their Relevance to Damping

Damping enhancement in epoxy composites is frequently accompanied by micro-scale energy absorption mechanisms (cavitation/shear yielding or interfacial friction), which also manifest in adhesion- and shear-dominated mechanical metrics [8,13,20,33]. Table 4 shows that CTBN significantly increases both lap-shear and peel strength (e.g., peel: 3.61 → 5.70 N/mm; shear: 12.98 → 17.39 MPa for EP → C25), consistent with improved ductility and higher shear energy absorption capacity [30,31,32,33].

In contrast, the h-BN-only formulation (B15) exhibits a modest reduction in peel strength (3.61 → 3.15 N/mm) despite a shear increase, suggesting that rigid platelets may introduce interface discontinuities or local stress concentrations when dispersion/compatibilization is not fully optimized. This observation is important because laminate-level damping is highly sensitive to interphase integrity; excessively weakened interfaces can compromise both load transfer and effective frictional dissipation. Therefore, a hybrid strategy is rational: CTBN can toughen/bridge interfaces while h-BN provides frictional losses, potentially mitigating the peel-strength penalty through improved interphase compliance [23,33,34,35].

### 3.5. Synergistic EPBN/h-BN Hybrid Matrix: Quantitative Verification

We benchmark the hybrid response against the linear-additive reference *tan δ_add* = *tan δ_max*, C + *tan δ_max*, B – *tan δ_max*, EP. Using the means from Table 3 (EP: 0.252; C25: 0.300; B25: 0.437), we can find that tan δ_add = 0.485. The hybrid (C25-B25) shows a *tan δ_max* = 0.468, corresponding to a synergy-efficiency index *η_tan δ* = 0.468/0.485 = 0.97, i.e., a near-additive response in *tan δ_max*. Given the reported experimental scatter (Table 3, *n* = 3), we therefore avoid claiming statistically significant above-additivity and instead describe the hybrid as achieving a high, near-additive damping peak while also delivering the broadest damping window and the best laminate-level vibration decay.

**Figure 5:**
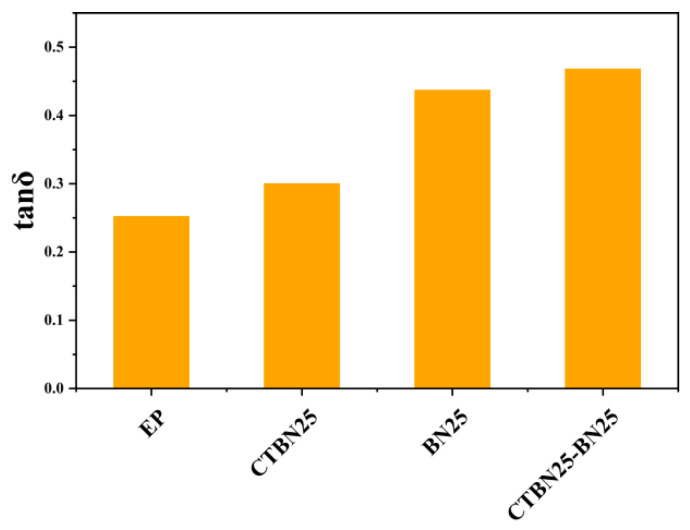
Synergistic damping performance of CTBN/h-BN hybrid epoxy: comparison of *tan δ_max*.

For *ΔT_half*, the linear-additive estimate is *ΔT_half*, add = 24.8 + 23.5 − 18.5 = 29.8 °C, and the hybrid gives 28.6 °C (*η_ΔT* = 0.96), which, again, is near-additive. For *T_tan δ*, we report absolute values and do not interpret small differences (<~2 °C) as meaningful without uncertainty bounds. As show in Figure 5, the main practical outcome is that the CTBN/h-BN hybrid simultaneously provides (i) a high *tan δ_max*, (ii) a broad *ΔT_half*, and (iii) improved laminate-level damping metrics rather than a strictly ‘synergistic’ deviation beyond additivity.

### 3.6. Damping Transfer from Matrix to CFRP Laminates

**Figure 6:**
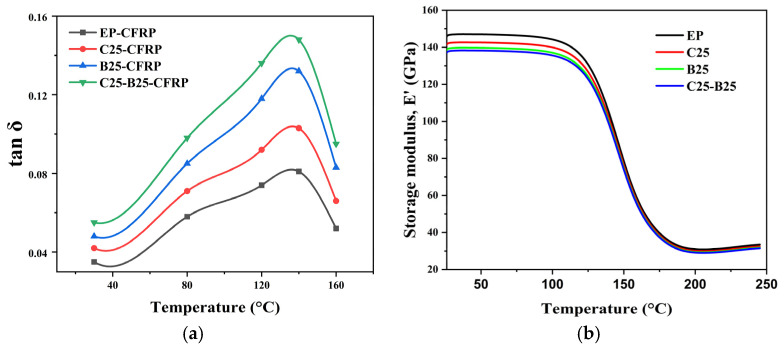
Dynamic mechanical analysis of CFRP laminates with different matrices: (**a**) loss factor *(tan δ*) and (**b**) storage modulus (E′) as a function of temperature.

Regarding the DMA response of the laminates, Figure 6 compares the temperature-dependent tan δ and storage modulus E′ of CFRP laminates using different matrices. We conducted a consistency check with UD-CFRP benchmark stiffness. For the 0° UD laminates (T700 12K UD CFRP), the room-temperature storage modulus E′ is ~138–145 GPa (25–80 °C) based on the DMA raw data, which falls in the expected fiber-dominated stiffness range for UD carbon/epoxy laminates and avoids nonphysical modulus deviations. Moreover, E′ remains nearly constant up to ~120 °C and then drops sharply as the matrix approaches its glass transition region; this “plateau-then-collapse” profile is consistent with the glassy-to-rubbery transition behavior of epoxy matrices in CFRPs. The hybrid matrix is expected to increase laminate-level *tan δ* while limiting the E′ penalty compared with a rubber-only modification, because h-BN introduces frictional losses without relying solely on soft elastomeric content [17,24,34,36].

**Figure 7:**
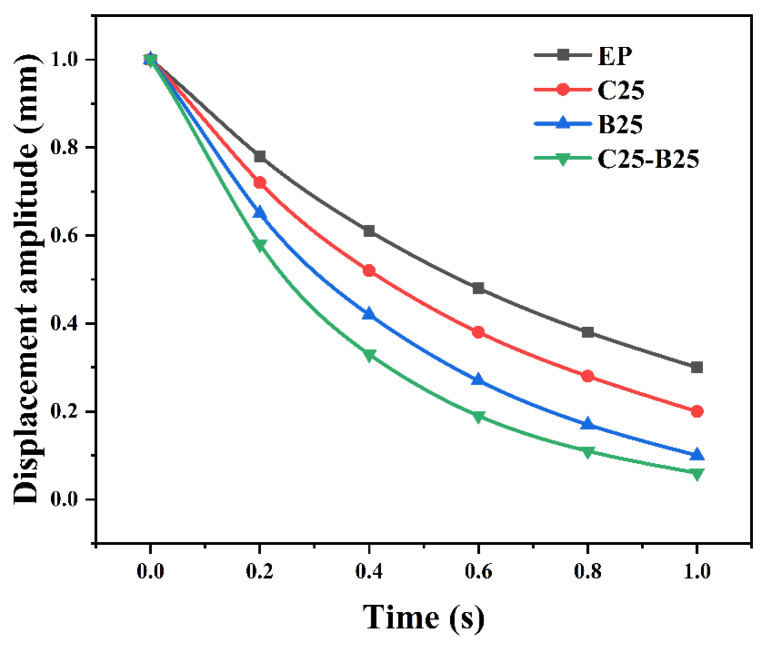
Free-vibration decay curves of CFRP cantilever beams with different matrices, showing enhanced structural damping for the CTBN/h-BN hybrid system.

Free-vibration damping: Figure 7 and Table 5 provide direct structural-level validation. Compared with EP-CFRP *(ζ* = 0.021; *δ* = 0.132; t_1_/_2_ = 0.48 s), the hybrid laminate CB25-25-CFRP achieves the highest damping *(ζ* = 0.035; *δ* = 0.221) and the fastest decay (t_1_/_2_ = 0.27 s). The CTBN-only and h-BN-only laminates show intermediate improvements (C25-CFRP: *ζ* = 0.026; B25-CFRP: *ζ* = 0.031), indicating that both ductile matrix dissipation and frictional sheet-mediated losses contribute to structural damping, with the hybrid delivering the most balanced enhancement. The use of a logarithmic decrement for *ζ* extraction is in line with standard underdamped vibration analysis practice [24,25]. For the 0° UD laminates, the room-temperature storage modulus is on the order of 140 GPa, which is consistent with the fiber-dominated stiffness expected for T700 12K UD CFRP and can serve as a benchmark for avoiding nonphysical modulus deviations.

## 4. Conclusions

An E51/DICY/accelerator epoxy system (100:6.5:1.2) was used as a baseline matrix for damping-oriented modification. CTBN increases epoxy damping with an optimum around 25 phr (*tan δ_max* = 0.300), accompanied by the expected stiffness/thermal trade-offs. h-BN provides a stronger monotonic tan δ enhancement up to 25 phr *(tanδ_max* = 0.437) with only modest changes in peak temperature. The CTBN/h-BN hybrid (25/25 phr) achieves a high, near-additive resin-stage damping response (*tanδ_max* = 0.468; *ΔT_half* = 28.6 °C), which translates into the highest laminate-level damping in free-vibration tests (*ζ*: 0.021 → 0.035), while maintaining a realistic 0° UD-CFRP storage modulus benchmark. Accordingly, the key contribution of this study is a processing-compatible hybrid-matrix design and a conservative additivity analysis reported with uncertainties rather than an arbitrary synergy threshold.

## Figures and Tables

**Table 1:** Resin and composite material formulation table.

Sample	CTBN (phr)	h-BN (phr)	Remark
EP	0	0	
C5	5	0	CTBN
C15	15	0	CTBN
C25	25	0	CTBN Optimal
B5	0	5	h-BN
B15	0	15	h-BN
B25	0	25	h-BN Optimal
C15-B15	15	15	Collaboration
C25-B15	25	15	Collaboration
C25-B25	25	25	Collaboration Optimal

**Table 2:** DSC characteristic parameters.

Sample	*Tp* (°C)	*ΔH* (J/g)	*Tg* (°C)
EP	160.6 ± 0.5	386.5 ± 8	133.4 ± 0.7
C15	164.2 ± 0.6	312.4 ± 6	129.6 ± 0.8
C25	167.4 ± 0.5	264.6 ± 5	126.8 ± 0.6
B15	165.8 ± 0.4	298.7 ± 7	135.2 ± 0.5
B25	168.9 ± 0.6	271.3 ± 6	137.6 ± 0.4
C25-B25	170.3 ± 0.5	255.1 ± 5	134.1 ± 0.6

**Table 3:** Key parameters of DMA (mean ± SD, *n* = 3).

Sample	*Tan δ_max*	*T_tanδ* (°C)	*ΔT_Half* (°C)
EP	0.252 ± 0.01	142.1	18.5
C5	0.273 ± 0.01	139.8	20.2
C15	0.289 ± 0.01	138.2	22.1
C25	0.300 ± 0.01	136.7	24.8
C35	0.291 ± 0.01	135.9	25.0
B15	0.389 ± 0.02	145.6	21.3
B25	0.437 ± 0.02	147.2	23.5
C25-B25	0.468 ± 0.02	143.8	28.6

**Table 4:** Interfacial mechanical properties.

Sample	Shear Strength (MPa)	Peel Strength (N/mm)
EP	12.98 ± 0.6	3.61 ± 0.2
C15	15.62 ± 0.5	4.83 ± 0.3
C25	17.39 ± 0.7	5.70 ± 0.3
B15	14.33 ± 0.4	3.15 ± 0.2
C25-B25	16.88 ± 0.5	5.12 ± 0.3

**Table 5:** CFRP damping parameters.

Sample	*ζ*	Logarithmic Decrement δ	Half-Life t_1_/_2_ (s)
EP-CFRP	0.021	0.132	0.48
C25-CFRP	0.026	0.163	0.39
B25-CFRP	0.031	0.194	0.32
CB25-25-CFRP	0.035	0.221	0.27

## Data Availability

The original contributions presented in the study are included in the article. Further inquiries can be directed to the corresponding authors.
